# Hyperpigmented mycosis fungoides: a rare variant

**DOI:** 10.11604/pamj.2013.15.13.2789

**Published:** 2013-05-07

**Authors:** Meryem Soughi, Fatima Zahra Mernissi

**Affiliations:** 1 CHU HAssan 2, Fes, Morocco

**Keywords:** mycosis fungoides, skin biopsy, photoprotection

## Image in medicine

Mycosis fungoides (MF) is the most common type of cutaneous T-cell lymphoma. The diagnosis of classic MF is based on a combination of clinical presentation, histopathology, immunohistochemistry, and T-cell monoclonality detected by molecular studies. However, the diagnosis can be difficult in some cases. We report a case of hyperpigmented mycosis fungoides. A 60-year-old woman, phototype IV, presented for more than 4 years a history of asymptomatic hyperpigmented non- infiltrated plaques on the face. Cutaneous lupus was diagnosed based on a skin biopsy. Hydroxychloroquine and photoprotection was started. Considering non improvement, another biopsy was done, in favor of a mycosis fungoides CD3+, CD4+, CD8-. The diagnostic of hyper pigmented mycosis fungoides stage I was retained and topical steroid was introduced. Mycosis fungoides can appear in various clinical forms, the hyperpigmented form is rare but this diagnosis should be evoked.

**Figure 1:**
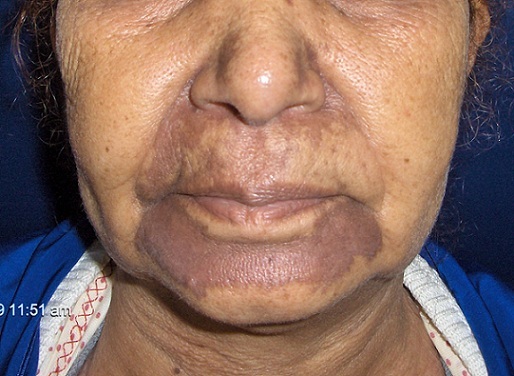
Pigmented plaques of the nasolabial fold and the chin

